# Light-responsive PyNAC90 orchestrates anthocyanin biosynthesis through combinatorial transcriptional activation and COP1-mediated degradation in pear

**DOI:** 10.1186/s43897-026-00254-7

**Published:** 2026-09-10

**Authors:** Chenhui Han, Zhaolong Xue, Guosong Chen, Yanfei Shan, Chenjie Yao, Yongqi Zhao, Bo Li, Yule Miao, Jiahao Zeng, Tianyu Wang, Guangyan Yang, Jun Wu

**Affiliations:** 1 College of Horticulture, National Key Laboratory of Crop Genetics & Germplasm Enhancement and Utilization, Nanjing Agricultural University, Nanjing, Jiangsu 210095 China; 2 Anhui Province Key Laboratory of Horticultural Crop Quality Biology, School of Horticulture, Anhui Agricultural University, Hefei, 230036 China; 3 Zhongshan Biological Breeding Laboratory, Nanjing, Jiangsu 210014 China

**Keywords:** Red-skinned pear, Anthocyanins, Light-induced, PyNAC90

## Abstract

Anthocyanins confer diverse biological activities and health benefits, and represent a key quality trait in fruit. In pear, red skin coloration arises from anthocyanin accumulation, a process strongly induced by light. Here, transcriptomic analyses identified PyNAC90 as a candidate regulator promoting anthocyanin biosynthesis. The expression level of *PyNAC90* gradually increased with extended bag removal. Stable transformation in pear callus and transient overexpression in pear skin showed that *PyNAC90* overexpression significantly enhanced anthocyanin accumulation, whereas *PyNAC90* silencing significantly suppressed anthocyanin biosynthesis. Dual-luciferase reporter assays demonstrated that PyHY5 directly induced *PyNAC90*, while PyNAC90 directly activated *PyMYB10* and indirectly activated the downstream structural genes *PyDFR* and *PyANS*. Furthermore, co-expression of PyNAC90, PyMYB10, and PybHLH3 synergistically activated the downstream structural genes *PyDFR* and *PyANS*. Moreover, Y2H, LCI, and BiFC assays confirmed that PyNAC90 physically interacted with PyMYB10, strengthening cooperative transcriptional regulation of anthocyanin biosynthesis. The E3 ubiquitin ligase PyCOP1 also interacted with PyNAC90 both in vivo and in vitro, mediating its degradation, thereby reducing anthocyanin accumulation. Collectively, our results elucidate a molecular mechanism by which light-responsive NAC transcription factors regulate anthocyanin biosynthesis and expand the regulatory network underlying light–induced pigmentation.

**Supplementary Information:**

The online version contains supplementary material available at https://doi.org/10.1186/s43897-026-00254-7.

## Core

Light induces the expression of *PyNAC90*, which is transcriptionally activated by PyHY5. PyNAC90 in turn directly activates the *PyMYB10* promoter and physically interacts with PyMYB10 to promote anthocyanin biosynthesis. Conversely, the E3 ubiquitin ligase PyCOP1 directly interacts with PyNAC90 and mediates its degradation, ultimately suppressing anthocyanin accumulation.

## Gene and accession numbers

Gene information is available at http://peargenome.njau.edu.cn/under the following accession numbers: *PyNAC90* (Pbr021741.1); *PyMYB10* (Pbr016663.1); *PyCOP1* (Pbr031957.1); *PybHLH3* (Pbr017379.1); *PyHY5* (Pbr002622.1).

## Introduction

Pears (*Pyrus* spp.) are a major commercial fruit crop in China (Ni et al. 2019). Based on skin color, cultivars are classified as white-, yellow-, green-, russet brown-, or red-skinned (Wu et al. 2013; Xue et al. 2017). Fruit skin color is a key determinant of commercial value in pear (Bai et al. 2019). A key determinant of coloration is anthocyanin accumulation, which confers strong antioxidant capacity, contributes to plant defense against pathogens and UV stress, and provides multiple health benefits to humans (Bieza and Lois 2001). Anthocyanin biosynthesis is driven by a cascade of structural genes whose expression is regulated by transcription factors. These genes are categorized into early biosynthetic genes (EBGs) and late biosynthetic genes (LBGs). LBGs, including dihydroflavonol 4-reductase (*DFR*), anthocyanin biosynthase (*ANS*), and UDP–glucose: flavonoid 3–glucosyltransferase (*UFGT*), are specifically dedicated to anthocyanin biosynthesis (Winkel-Shirley 2001).

Expression of anthocyanin structural genes is controlled by MBW complexes composed of R2R3-MYB, basic helix-loop-helix (bHLH), and WD40-repeat proteins (Wei et al. 2023). R2R3-MYB proteins act as core components that directly bind the promoters of anthocyanin biosynthetic genes, although regulatory mechanisms vary among species (Li et al. 2020). In apple, anthocyanin biosynthesis is enhanced through complex formation between MdMYB10 and its partners MdbHLH3 and MdbHLH33 (Espley et al. 2007). In pear, PyMYB107 competes with PyMYB10/MYB114 for binding to PybHLH3, thereby repressing transcription of key anthocyanin biosynthetic genes (Yang et al. 2024). Beyond MBW components, several transcription factor families, including NAC, ERF, bZIP, BBX, and WRKY, modulate anthocyanin biosynthesis through direct or indirect interactions with the MBW complex (Amato et al. 2017; Chang et al. 2023; Li et al. 2020; Yang et al. 2024; Zhou et al. 2015).

NAC proteins constitute one of the largest transcription factor families (NAM, ATAF1/2, and CUC2) and are characterized by a highly conserved N-terminal NAC domain of approximately 150 amino acids (Ooka et al. 2003). In contrast, their C-terminal regions function as transcriptional regulatory domains and exhibit substantial sequence diversity (Ooka et al. 2003). NAC transcription factors regulate diverse aspects of plant growth and development, including stress responses, lateral root formation, floral organ development, and fruit development and ripening (Zhang et al. 2020). Recent studies have confirmed NAC involvement in anthocyanin biosynthesis across multiple species, including apple (Sun et al. 2019), peach (Zhou et al. 2015), pear (Zhang et al. 2024), Tibetan Hulless Barley (Yao et al. 2022), and litchi (Jiang et al. 2019).

Anthocyanin biosynthesis is also strongly influenced by environmental signals (Shi et al. 2023), with light serving as a dominant regulator of both plant development and pigmentation across species (Takos et al. 2006). In apple, the *MdMYB16/MdMYB1*-*miR7125*-*MdCCR* module controls anthocyanin homeostasis under light induction (Hu et al. 2021). Several light-responsive transcription factors and signaling components, including HY5, COP1, and bZIPa, regulate anthocyanin biosynthesis by controlling expression of key enzymatic genes (An et al. 2017; Liu et al. 2019; Maier et al. 2013). LONG HYPOCOTYL 5 (HY5) functions as a central transcription factor downstream of CONSTITUTIVELY PHOTOMORPHOGENIC 1 (COP1) in light signaling (Bursch et al. 2020). COP1 and HY5 form a core antagonistic module that coordinates anthocyanin accumulation, whereas in darkness this process is repressed through PpCOP1-mediated degradation of *PpHYH* (Zhao et al. 2022). In eggplant, blue light inhibits *SmCOP1* via cryptochromes, and SmCOP1 directly regulates *SmMYB1*, suggesting that SmCOP1 represses anthocyanin biosynthesis through multiple pathways, potentially by targeting SmHY5 and other regulators for degradation (Jiang et al. 2016).

Although NAC transcription factors have been implicated in anthocyanin regulation, their specific roles within light-induced anthocyanin biosynthetic pathways in red pear skin remain poorly understood. In this study, we show that light-induced *PyNAC90* is activated by PyHY5 and that PyNAC90, in turn, directly activates the *PyMYB10* promoter and interacts with PyMYB10 to promote anthocyanin biosynthesis. PyNAC90 also interacts with PyCOP1, and under dark conditions, this interaction attenuates transcriptional activation of *PyDFR* and *PyANS*. We therefore propose that PyCOP1 destabilizes the PyNAC90 protein. These findings deepen our understanding of light-responsive NAC transcription factor function and provide a molecular framework for improving coloration and visual quality in red pear fruit.

## Results

### Expression of *PyNAC90* correlates with anthocyanin accumulation in red–skinned pear

Previous studies have shown that light strongly induces anthocyanin biosynthesis in pear. To expand the regulatory network underlying light-responsive anthocyanin accumulation, we conducted a comprehensive analysis of transcriptomic data from published fruit bagged and debagged experiments (Liu et al. 2019). Weighted gene co–expression network analysis (WGCNA) grouped expressed genes into 10 modules, among which the antiquewhite2 and darkslateblue modules were significantly associated with anthocyanin biosynthesis and were enriched in NAC, WRKY, ERF, and bHLH transcription factors (Figs. S1A–C). Differentially expressed gene (DEG) analysis was then performed (Fig. S1D), and integration of DEGs with key WGCNA modules (Fig. S1E) identified PyNAC90 as the most strongly differentially expressed transcription factor within its module. PyNAC90 was therefore selected as a candidate regulator of anthocyanin biosynthesis and a potential participant in light signaling (Liu et al. 2019). In contrast, its *Arabidopsis* homolog, AtNAC90, functions as a negative regulator of immune responses.

**Fig. 1:**
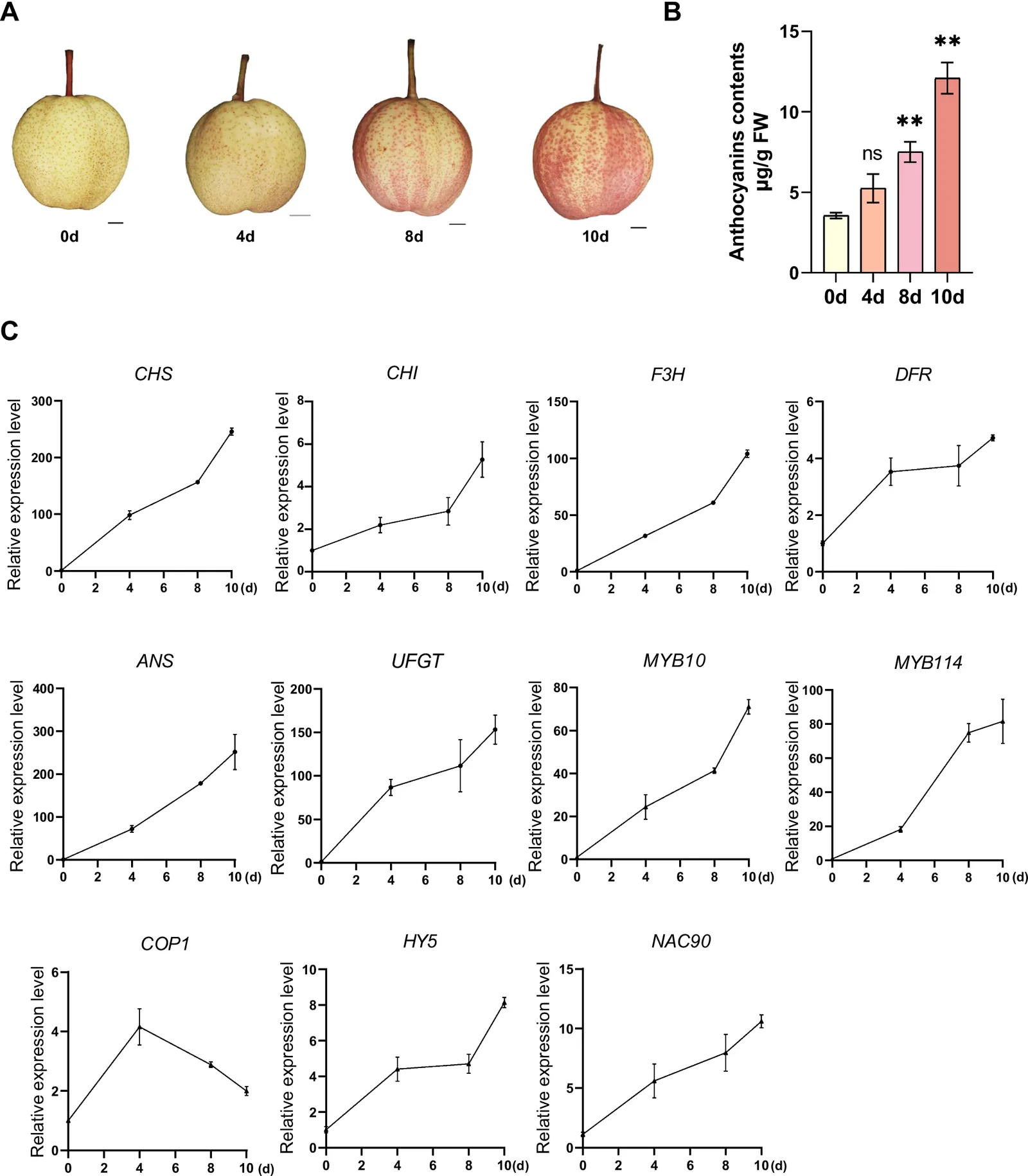
Color phenotype of pear fruits and expression levels of anthocyanin-related genes. **A** Light-induced phenotypes of ‘Red Zaosu’ pear fruit. Among the treatments, 0 d represents bagged fruit without light exposure, while 4 d, 8 d, and 10 d indicate fruit exposed to light for 4, 8, and 10 days, respectively. **B** Anthocyanin concentration in pear fruit skins. FW, fresh weight. **C** Expression patterns of genes associated with anthocyanin biosynthesis under different photoperiod regimes. The x-axis represents the duration of daylight exposure (days). Error bars denote mean ± SE of three biological replicates. ns: not significant; asterisks indicate significant differences as determined by Student’s *t*-test (** *P* < 0.01)

To characterize the light-responsive expression pattern of *PyNAC90*, ‘Red Zaosu’ pear fruits were subjected to three lighting regimes. Fruits in the control group (0 d) were bagged, whereas experimental groups were exposed to light for 4, 8, or 10 days after bag removal. With increasing duration of light exposure, epidermal pigmentation intensified, accompanied by a progressive increase in anthocyanin content (Fig. 1A and B). Consistent with these phenotypes, expression of major structural genes in the anthocyanin biosynthetic pathway, (*PyCHS*, *PyCHI*, *PyF3H*, *PyDFR*, *PyANS*, and *PyUFGT*) was significantly higher in the skins of debagged fruits than in bagged controls (Fig. 1C). Similarly, major regulatory genes, including *PyMYB10*, *PyMYB114*, and *PyHY5*, exhibited parallel expression patterns (Fig. 1C). Notably, *PyNAC90* expression was markedly elevated in debagged fruit skin and closely mirrored the expression profiles of core anthocyanin biosynthetic genes and regulators such as PyMYB10, suggesting that *PyNAC90* plays a role in light-induced anthocyanin biosynthesis in pear.

**Fig. 2:**
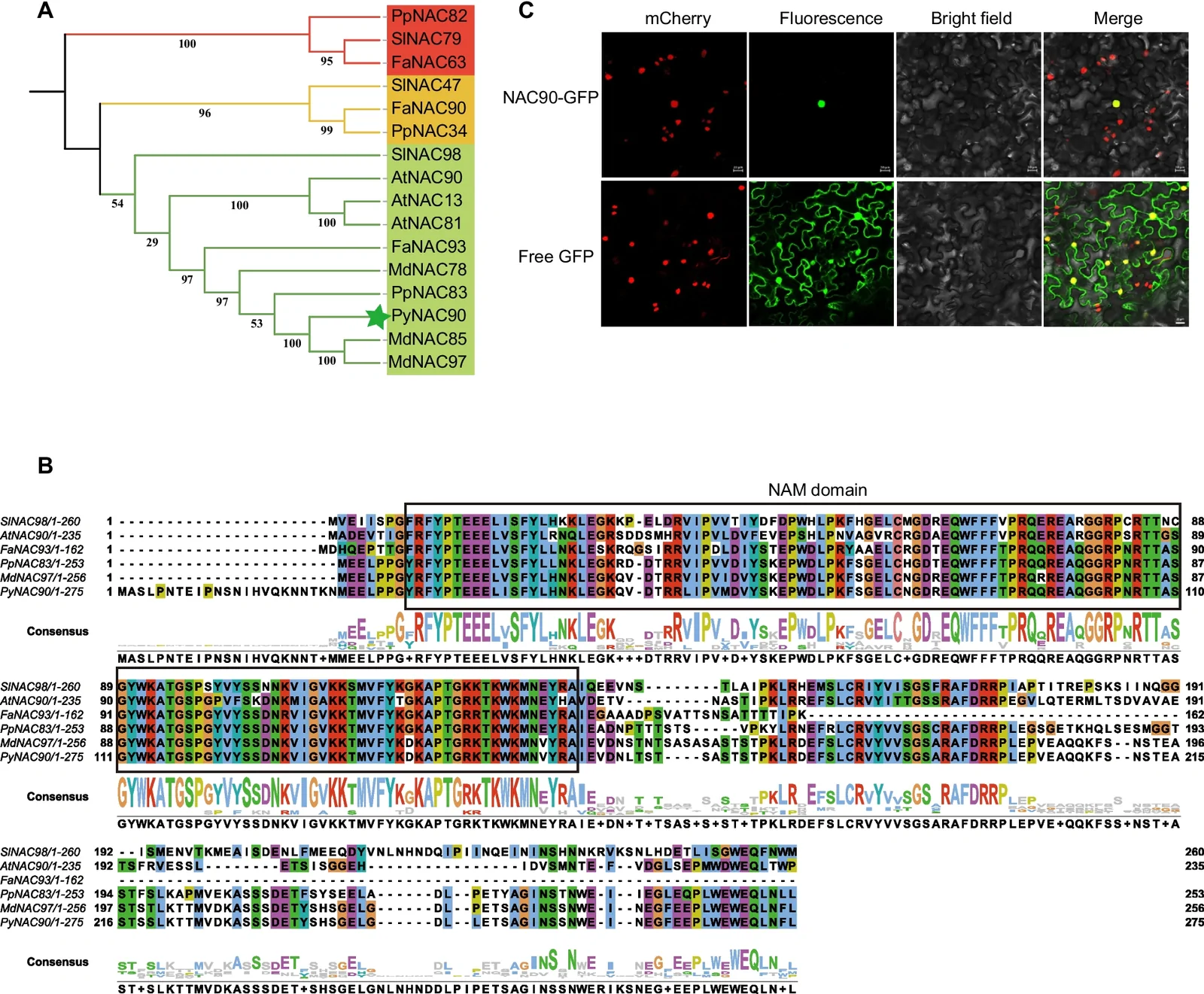
PyNAC90 protein and subcellular localization. **A** Neighbor-joining phylogenetic tree of PyNAC90 and its homologous genes from other species. **B** Amino acid sequence alignment of PyNAC90, PpNAC83, AtNAC90, MdNAC97, SlNAC98, and FaNAC93. The NAM domains are underlined. **C** PyNAC90 is a nuclear-localized protein, as detected by laser confocal microscopy. Scale bar = 20 μm

PyNAC90 encodes a 276–amino–acid protein containing a highly conserved NAM (no apical meristem) domain characteristic of NAC transcription factors. Phylogenetic analysis showed that PyNAC90 clusters closely with MdNAC85 and MdNAC97 (Fig. 2A). However, the roles of these apple NACs in anthocyanin biosynthesis remain uncharacterized. The NAM domain of PyNAC90 is highly conserved among Arabidopsis and multiple Rosaceae species (Fig. 2B). Subcellular localization analysis revealed that free GFP was distributed in both the nucleus and cytoplasm, whereas the PyNAC90-GFP fusion protein localized exclusively to the nucleus, confirming its nuclear accumulation (Fig. 2C).

### Overexpression of *PyNAC90* enhances anthocyanin biosynthesis in pear fruit and callus

**Fig. 3:**
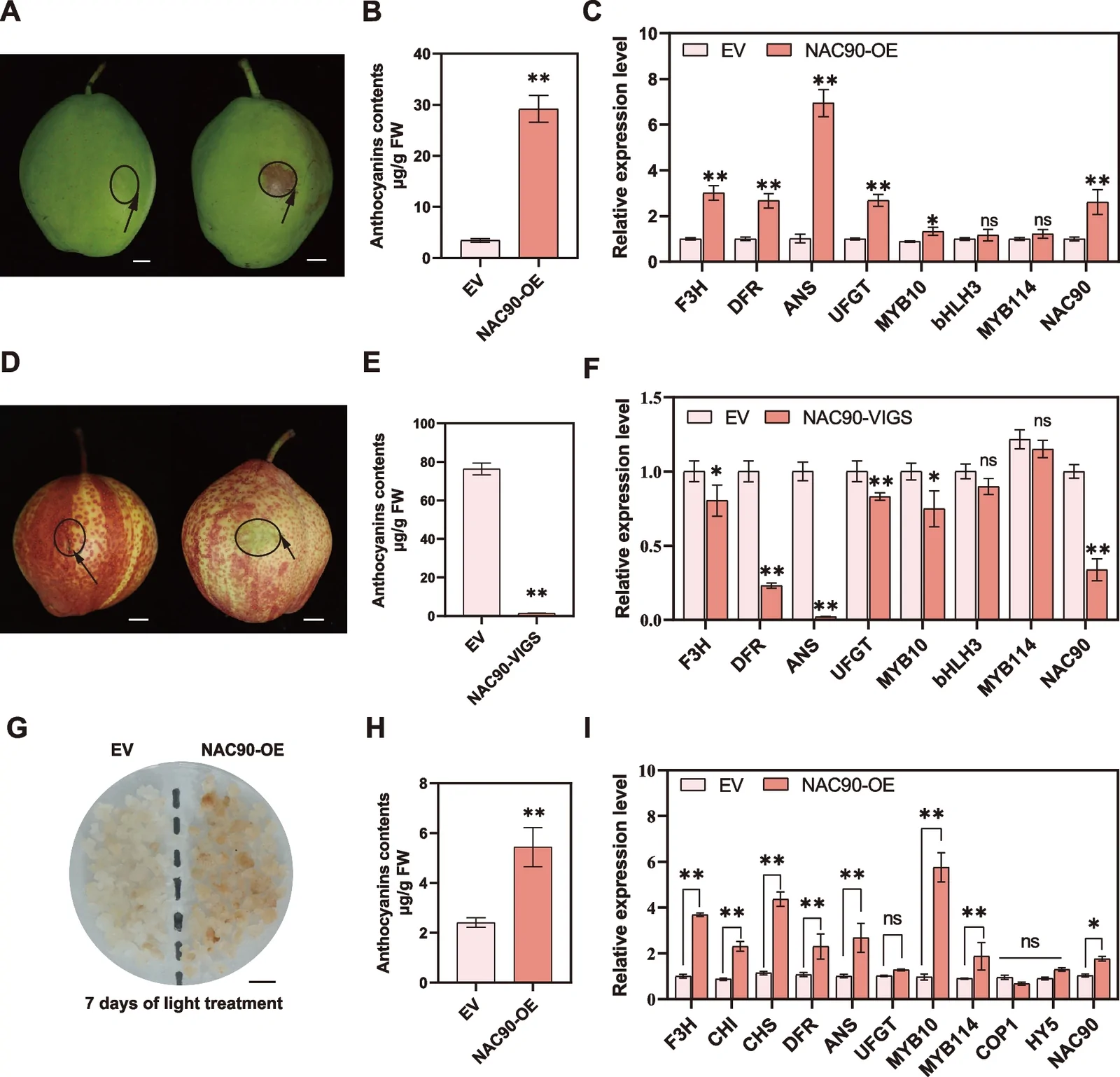
Transient expression and stable transformation of *PyNAC90* in pear fruits.** A** Transient overexpression of *PyNAC90* in ‘Zaosu’ pear fruit. EV (empty vector pSAK277) was used as the control. OE, overexpression. The scale bar in the figure represents 1 cm. Images were taken at 7 days post-agroinfiltration. The infiltrated area was outlined with a solid black circle. **B** Anthocyanin content in fruit skins of control and PyNAC90-OE fruit, measured by spectrophotometry. FW: fresh weight.** C** Expression levels of anthocyanin-related genes in control and PyNAC90-OE fruit skin, as analyzed by RT-qPCR. **D** Transient silencing of *PyNAC90* in ‘Red Zaosu’ pear fruit. EV: empty vector (TRV1 + TRV2) control; VIGS: virus-induced gene silencing. The scale bar in the figure represents 1 cm. **E** Anthocyanin content in the skin of EV and PyNAC90-VIGS fruit.** F** Expression levels of anthocyanin-related genes in EV and PyNAC90-VIGS fruit skin.** G** Overexpression of PyNAC90 induced anthocyanin accumulation in pear calli. The calli transformed with the empty vector (pCAMBIA1301) were used as the negative control. Pear calli were incubated at 17 °C for 7 days. The scale bar in the figure represents 1 cm. **H** Anthocyanin content in *PyNAC90*-OE pear calli after light treatment.** I** Expression levels of anthocyanin-related genes and PyNAC90 in *PyNAC90*-OE pear calli after light treatment. Error bars denote mean ± SE of three biological replicates. ns: not significant; asterisks indicate significant differences as determined by Student’s *t*-test (* *P* < 0.05, ** *P* < 0.01)

To determine the functional role of *PyNAC90*, the overexpression construct 35S::PyNAC90 (PyNAC90-OE) and a virus-induced gene silencing construct (PyNAC90-VIGS) were introduced into ‘Zaosu’ and ‘Red Zaosu’ pear fruit via agroinfiltration. Seven days after infiltration, *PyNAC90* overexpression significantly increased anthocyanin accumulation relative to the empty vector control (Fig. 3A and B), accompanied by upregulated expression of main anthocyanin biosynthetic related genes, including *PyANS*, *PyUFGT*, and *PyMYB10* (Fig. 3C). *PyNAC90* silencing reduced pigmentation and anthocyanin content compared with controls (Fig. 3D and E), and expression of major anthocyanin biosynthetic genes was correspondingly downregulated (Fig. 3F). Furthermore, to verify whether *PyNAC90* is functional under dark conditions, the samples were subjected to dark treatment for 24 h after transient injection, after which *PyNAC90* function was assessed. The results showed that *PyNAC90* does not promote anthocyanin biosynthesis in the dark (Fig. S4).

Stable overexpression of *PyNAC90* in pear calli also enhanced anthocyanin biosynthesis under light conditions relative to empty vector (Fig. 3G and H). Consistently, transcript levels of major structural genes (*PyCHS*, *PyCHI*, *PyF3H*, *PyDFR*, and *PyANS*) and regulatory genes (*PyMYB10* and *PyMYB114*) were significantly elevated in PyNAC90-overexpressing calli compared with wild-type controls (Fig. 3I).

### *PyNAC90* is activated by PyHY5 and promotes PyMYB10-dependent activation of anthocyanin structural genes

**Fig. 4:**
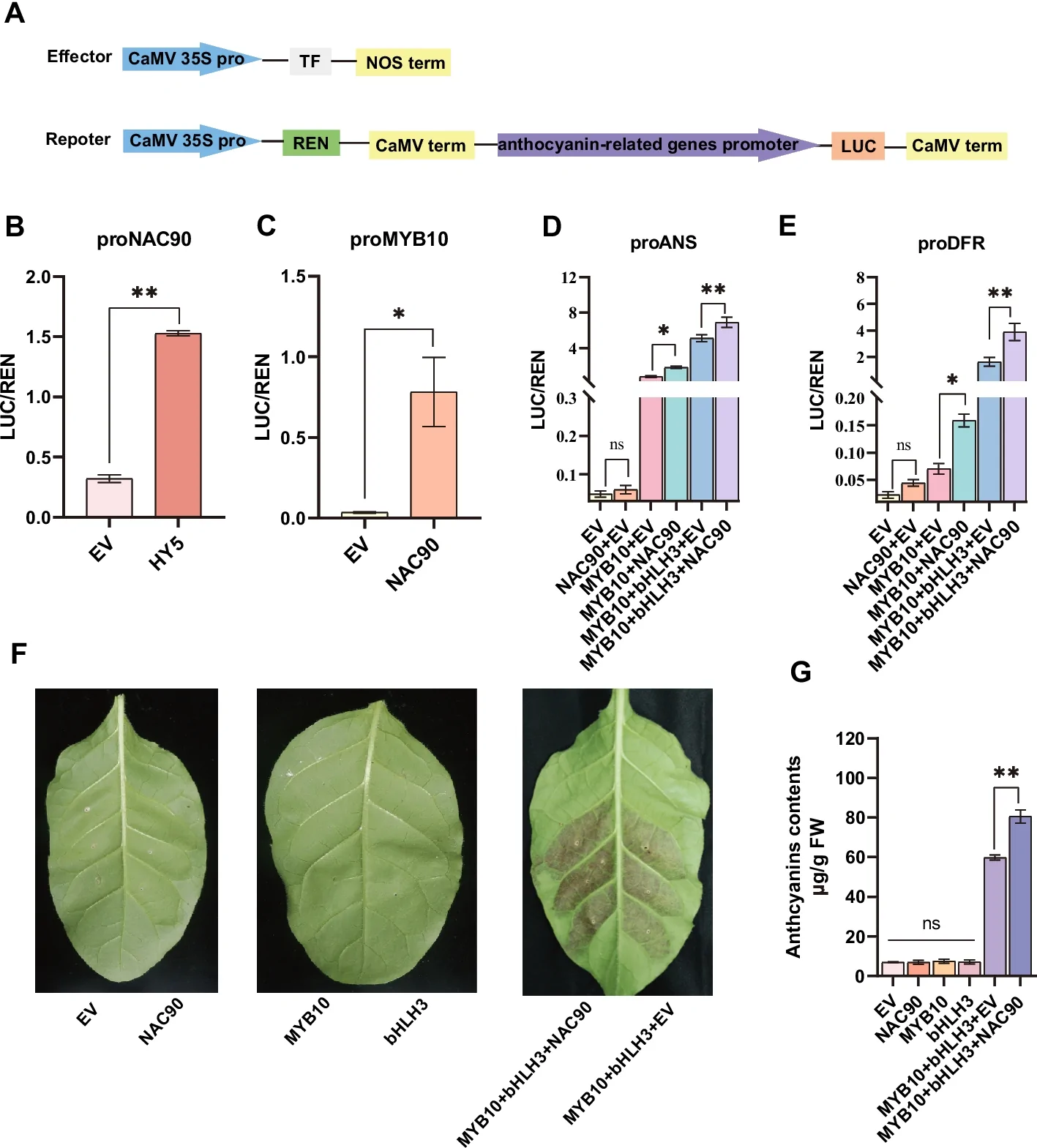
*PyNAC90* is activated by PyHY5 and promotes PyMYB10-dependent activation of anthocyanin biosynthetic structural genes. **A** Schematic representation of the effector and reporter constructs used in the dual-luciferase assay. **B** PyHY5 directly induces *PyNAC90* transcription in the dual-luciferase assay. **C**–**E** Dual-luciferase assay detecting PyNAC90-mediated transactivation of the *PyMYB10*, *PyDFR*, and *PyANS* promoters. **F** Transient color assay of PyNAC90 activity in tobacco leaves. Images were taken at 7 days post-inoculation. **G** Anthocyanin accumulation following transient injection in ‘Samsun’ tobacco (*Nicotiana tabacum*). Error bars denote mean ± SE of three biological replicates. Asterisks indicate statistically significant differences as determined by Student’s *t*-test (* *P* < 0.05, ** *P* < 0.01)

To elucidate the regulatory mechanism of *PyNAC90*, we performed dual-luciferase reporter assays. The *PyNAC90* promoter was fused to a LUC reporter (Fig. 4A), and high luciferase activity indicated that PyHY5 directly activates the *PyNAC90* promoter (Fig. 4B). But, the yeast one-hybrid assay showed that PyHY5 does not bind to the PyNAC90 promoter (Fig. S2F). Promoters of the key structural genes *PyDFR* and *PyANS* and the regulatory gene *PyMYB10* were then cloned to test whether PyNAC90 could activate their transcription (Fig. 4A). PyNAC90 alone did not directly activate *PyDFR* or *PyANS* but significantly activated the *PyMYB10* promoter directly (Fig. 4C). By contrast, PyMYB10, either alone or together with PybHLH3, activated both structural gene promoters, and co-expression with PyNAC90 further enhanced this activation (Fig. 4D and E), indicating a synergistic role of *PyNAC90* in promoting anthocyanin biosynthesis.

Yeast one-hybrid (Y1H) assays showed that PyNAC90 does not directly bind the promoters of *PyMYB10*, *PyDFR*, or *PyANS* (Fig. S2C–E), suggesting that its regulatory effects are mediated through interactions with other transcriptional regulators. To test whether PyNAC90 enhances anthocyanin accumulation in cooperation with PyMYB10 and PybHLH3, we transiently expressed these genes in *Nicotiana tabacum* (Fig. 4F). As previously reported, co-infiltration of PyMYB10 and PybHLH3 significantly increased anthocyanin levels (Ni et al. 2023; Yao et al. 2017; Zhai et al. 2016). Importantly, inclusion of PyNAC90 further amplified anthocyanin accumulation compared with PyMYB10 and PybHLH3 alone (Fig. 4F and G), indicating that PyNAC90 promotes anthocyanin biosynthesis by cooperating with PyMYB10 and PybHLH3, likely as part of a ternary regulatory complex.

### PyNAC90 physically interacted with PyMYB10

**Fig. 5:**
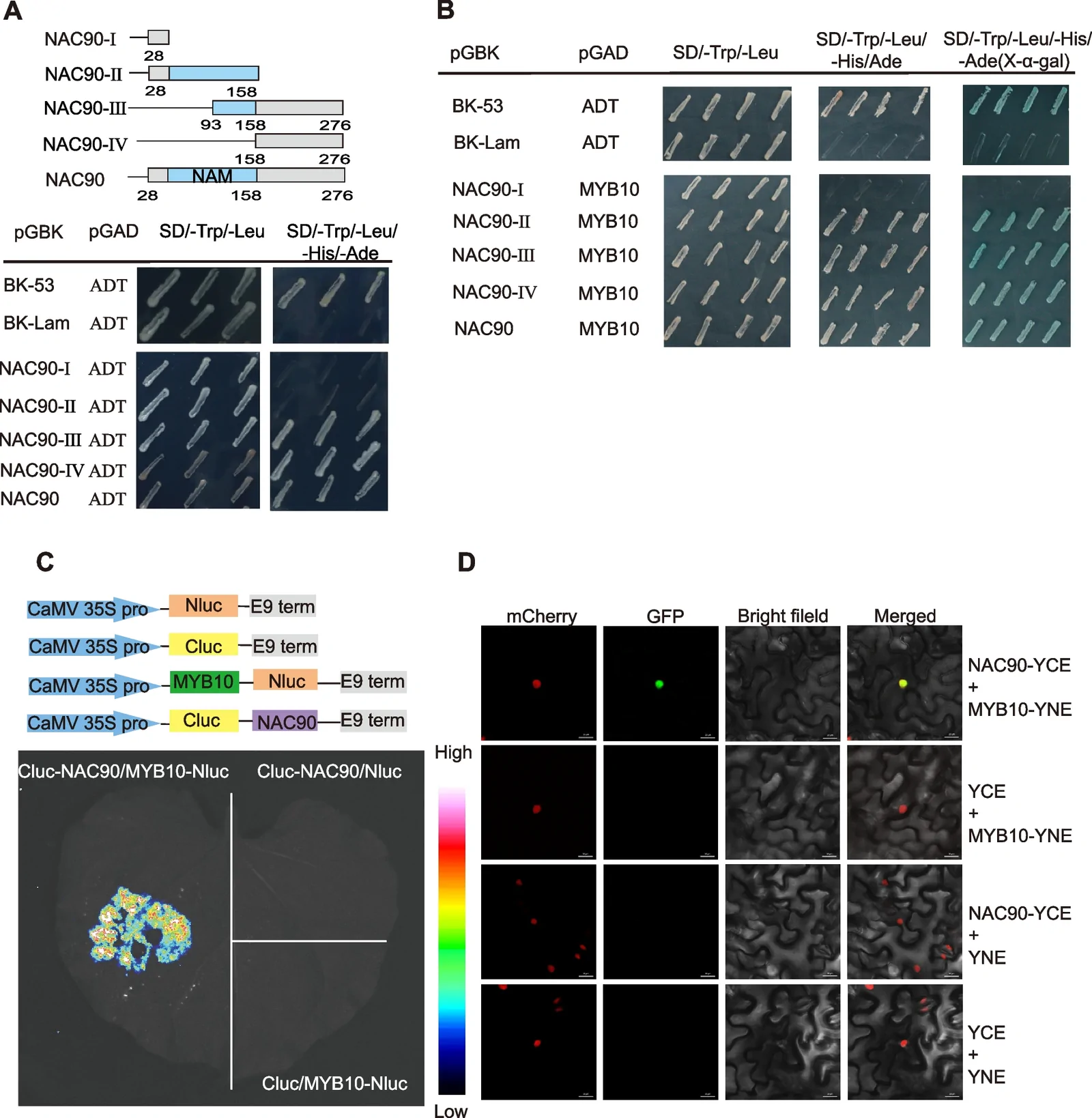
PyNAC90 interacts with PyMYB10. **A** Autoactivation assay of full-length and truncated PyNAC90. PyNAC90-I and PyNAC90-II showed no self-activation. **B** Yeast two-hybrid assay verifying the interaction between PyMYB10 and PyNAC90, as well as its truncated variants. **C** Interaction between PyNAC90 and PyMYB10 was detected by firefly luciferase complementation imaging in *N. benthamiana* leaves. The color bar (blue to red) corresponds to luminescence intensity. **D** Bimolecular fluorescence complementation (BiFC) assay. Scale bar = 20 μm

Although PyNAC90 acts upstream of *PyMYB10*, whether the two proteins physically interact remained unclear. To address this, we performed yeast two-hybrid (Y2H) assays. *PyNAC90* was fused to the GAL4 DNA-binding domain (BD) as bait, while *PyMYB10*, *PyMYB114*, and *PybHLH3* were individually fused to the GAL4 activation domain (AD) as prey. Because bait autoactivation can confound Y2H analyses, we first tested whether PyNAC90-BD activated reporter genes autonomously. Yeast co-transformed with PyNAC90-BD and the empty AD vector grew on both SD/-Trp/-Leu and SD/-Trp/-Leu/-Ade/-His media (Fig. 5A), indicating self-activation. To eliminate this effect, PyNAC90 was truncated into four fragments (PyNAC90-I, PyNAC90-II, PyNAC90-III, and PyNAC90-IV). Among these, PyNAC90-I and PyNAC90-II showed no self-activation, whereas PyNAC90-III and PyNAC90-IV retained self-activating activity (Fig. 5A). Using the non-self-activating fragments, we found that the full NAM domain of PyNAC90 interacted with PyMYB10 (Fig. 5B). No interaction was detected between PyNAC90 and PyMYB114 or PybHLH3 (Fig. S2A and B).

These interactions were further validated in planta. In the firefly luciferase complementation imaging (LCI) assay, co-expression of PyNAC90-Cluc and PyMYB10-Nluc produced a strong luminescent signal, confirming their physical interaction in vivo (Fig. 5C). Consistently, bimolecular fluorescence complementation (BiFC) assays in *N. benthamiana* leaves revealed strong nuclear yellow fluorescence upon co-expression of PyNAC90-YNE and PyMYB10-YCE (Fig. 5D). Together, these results demonstrate that PyNAC90 physically interacts with PyMYB10.

### PyNAC90 activity is suppressed by the light signaling repressor PyCOP1

**Fig. 6:**
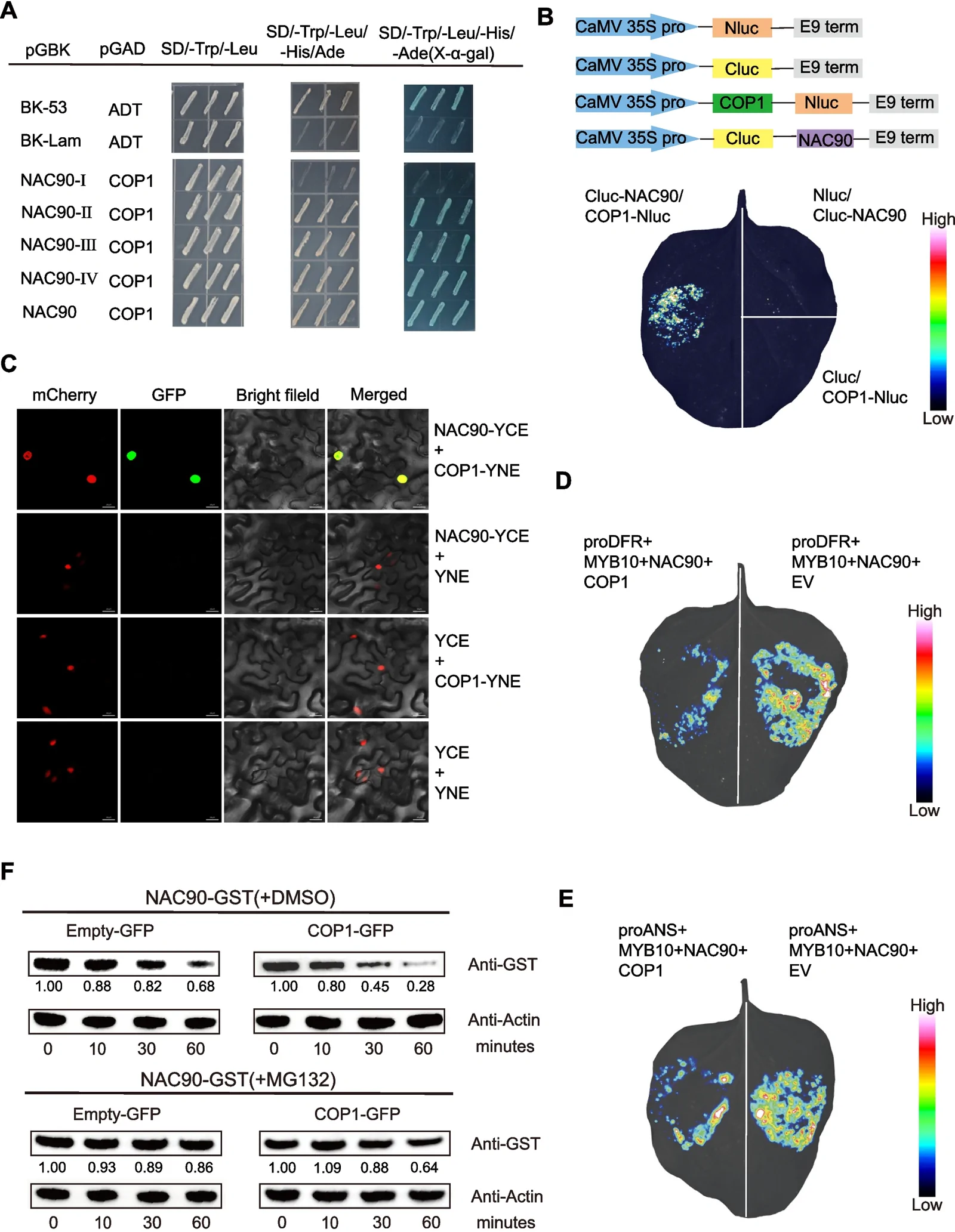
PyNAC90 interacts with PyCOP1. **A** Yeast two-hybrid assay verifying the interaction between PyCOP1 and the four-segment truncation variant PyNAC90. **B** Firefly luciferase complementation imaging (LCI) was used to evaluate the interaction between PyNAC90 and PyCOP1. The color bar (blue to red) corresponds to luminescence intensity. **C** Bimolecular fluorescence complementation (BiFC) assay. Scale bar = 50 μm. **D–E** The interaction between PyCOP1 and PyNAC90 leads to attenuated transcriptional activation of *PyANS* and *PyDFR*. **F** Cell-free degradation assay of PyNAC90-GST. Total proteins were extracted from *N. benthamiana* leaves transiently expressing empty GFP and PyCOP1-GFP. The protein extraction solution was treated with 100 μM DMSO and MG132 for 30 min, then incubated with recombinant PyNAC90-GST for the indicated times. PyNAC90-GST protein levels were detected using an anti-GST antibody. Actin protein levels were used as a control

To determine how PyNAC90 integrates light signals during pear skin coloration, we conducted a yeast two-hybrid screen and identified the E3 ubiquitin ligase PyCOP1 as a PyNAC90–interacting protein (Fig. 6A). This interaction was confirmed by luciferase complementation imaging (LCI) (Fig. 6B) and further validated by BiFC assays in living *N. benthamiana* cells (Fig. 6C). These interactions suggest that PyCOP1 mediates PyNAC90 degradation. Consistently, dual-luciferase assays showed that co-expression of PyMYB10, PyNAC90, and PyCOP1 significantly reduced the transcriptional activity of the *PyANS* and *PyDFR* promoters, demonstrating that PyCOP1 suppresses PyNAC90 protein activity (Fig. 6D and E). We also performed cell-free degradation assays using purified recombinant PyNAC90-GST protein. PyCOP1-GFP and GFP empty proteins were expressed in the leaves of *N. benthamiana*. Subsequently, Western blot analysis showed that PyNAC90-GST degraded over time in GFP and PyCOP1-GFP transgenic samples (Fig. 6F). Importantly, compared to GFP, the degradation of PyNAC90-GST is faster in the presence of PyCOP1-GFP protein. The addition of MG132 effectively inhibited PyCOP1-induced degradation of the PyNAC90 protein, confirming the involvement of the 26S proteasome in this process. These results indicated that PyCOP1 can degrade PyNAC90.

## Discussion

### Light-induced PyNAC90 functions as a positive regulator of anthocyanin biosynthesis

NAC transcription factors act as central integrators of developmental, stress, and metabolic signaling pathways (Olsen et al. 2005; Zhang et al. 2020). For example, MdNAC52 enhances anthocyanin accumulation in apple by directly activating *MdMYB9* and *MdMYB11* (Sun et al. 2019). In peach, NACs regulate fruit ripening, sugar accumulation, and growth (Dai et al. 2023; Zhang et al. 2025). Similarly, promoter analyses in *Citrullus colocynthis* revealed light-responsive motifs in *CcNAC1* and *CcNAC2*, suggesting their roles in light signal transduction (Wang et al. 2014). Consistent with these reports, *PyNAC90* expression in pear skin is strongly induced by light, with higher expression following bag removal compared with bagged fruits, confirming its light responsiveness (Wang et al. 2014). Furthermore, its expression level is low under dark conditions, with no significant difference observed (Fig. S3). Promoter analysis of *PyNAC90* identified multiple light-responsive cis-elements (Tab. S1), and our experiments demonstrate that although PyHY5 does not directly bind the PyNAC90 promoter (Fig. S2F), it directly activates *PyNAC90* transcription, placing *PyNAC90* downstream of the canonical light signaling pathway (Fig. 4B).

While NACs in other species, such as PpNAC22 and PpNAC100 in peach mesocarp (Zhou et al. 2015), act as transcriptional activators of MYB genes to promote anthocyanin biosynthesis through homodimerization (Liang et al. 2025), PyNAC90 exhibits functional divergence from its *Arabidopsis* ortholog AtNAC90, which negatively regulates immune responses. In contrast, *PyNAC90* positively regulates anthocyanin biosynthesis in pear fruit skin. Overexpression of *PyNAC90* in pear calli, fruit skin, and tobacco significantly enhanced anthocyanin biosynthesis, whereas transient silencing reduced pigmentation (Figs. 3 and 4). RT–qPCR analysis confirmed that *PyNAC90* overexpression upregulates anthocyanin biosynthetic genes (*PyCHS*, *PyCHI*, *PyF3H*, *PyDFR*, *PyANS*, and *PyUFGT*), consistent with its role as a positive regulator (Fig. 3).

### PyNAC90 and PyMYB10 cooperate synergistically to enhance anthocyanin biosynthesis

MYB transcription factors are central regulators of anthocyanin structural genes (Chagné et al. 2013). In pear, both PyMYB10 and PyMYB114 promote anthocyanin accumulation by directly activating target gene promoters (Feng et al. 2010; Yao et al. 2017; Tao et al. 2018). MYB factors are also modulated by other transcriptional regulators, forming hierarchical modules. For example, PpBBX16 activates the *PpMYB10* promoter (Bai et al. 2019), whereas PpWRKY26 enhances *PpMYB114* promoter activity (Li et al. 2020).

Although PyNAC90 does not directly bind the *PyMYB10* promoter (Fig. S2C), it directly activates the promoter of *PyMYB10*, increasing the expression of downstream anthocyanin biosynthetic genes (*PyDFR* and *PyANS*) and promoting pigment accumulation (Fig. 4). NAC transcription factors, including PyNAC90, exhibit autoactivation potential (Jia et al. 2018), likely amplifying downstream transcription and enhancing anthocyanin biosynthesis (Liu et al. 2023). Both in vitro and in vivo assays confirmed that PyNAC90 physically interacts with PyMYB10 (Fig. 5). Co-expression of PyNAC90 and PyMYB10 significantly increased *PyANS* and *PyDFR* promoter activity relative to PyMYB10 alone. Furthermore, the ternary PyNAC90–PyMYB10–PybHLH3 complex enhanced promoter activation beyond the MYB–bHLH dimer alone (Fig. 4D and E), demonstrating that PyNAC90 potentiates the MBW complex to promote anthocyanin accumulation in pear skin.

However, PyNAC90 interacts with PyMYB10 but not with PyMYB114 (Fig. S1A). Actually, *PyMYB114* and *PyMYB10* are both reported regulating anthocyanin biosynthesis in pear skin (Bai et al. 2019; Li et al. 2020; Premathilake et al. 2020; Yao et al. 2023). Anthocyanin biosynthesis is a complex regulatory network, *PyMYB10* and *PyMYB114* play different functions due to different response signals. Protein sequence comparison showed that PyMYB10 and PyMYB114 share 43.78% sequence identity and exhibit obvious differences in their C-terminal regions. Specifically, the C-terminus of PyMYB10 contains a DCI domain and an acidic amino acid-rich region, whereas these features are absent in PyMYB114. Acidic amino acids, which participate in electrostatic interactions between proteins, act as a “glue” for protein interaction networks; their replacement has been shown to disrupt such interactions (O'Shea et al. 2015). Therefore, we speculate that the structural differences between the two proteins underlie their interaction specificity with PyNAC90. 

### PyCOP1 mediates PyNAC90 degradation to modulate light-responsive anthocyanin biosynthesis

COP1 is a central repressor of photomorphogenesis (Bursch et al. 2020), mediating the ubiquitin-dependent degradation of positive light-signaling regulators in darkness (Hoecker 2017). In *Arabidopsis*, AtCOP1 controls light-induced anthocyanin biosynthesis by modulating AtMYB75 stability (Li et al. 2016), while in tomato, SlCOP1 regulates SlHY5 stability to integrate light signaling into anthocyanin biosynthesis (Liu et al. 2018). Light-mediated inactivation of E3 ligases, such as LvCOP1 in lily, prevents degradation of anthocyanin activators, enabling transcriptional activation of structural genes and pigmentation (Sun et al. 2024). Similarly, MdBT2 promotes COP1 accumulation in apple, enhancing degradation of MYB activators and suppressing anthocyanin synthesis (Kang et al. 2022).

**Fig. 7:**
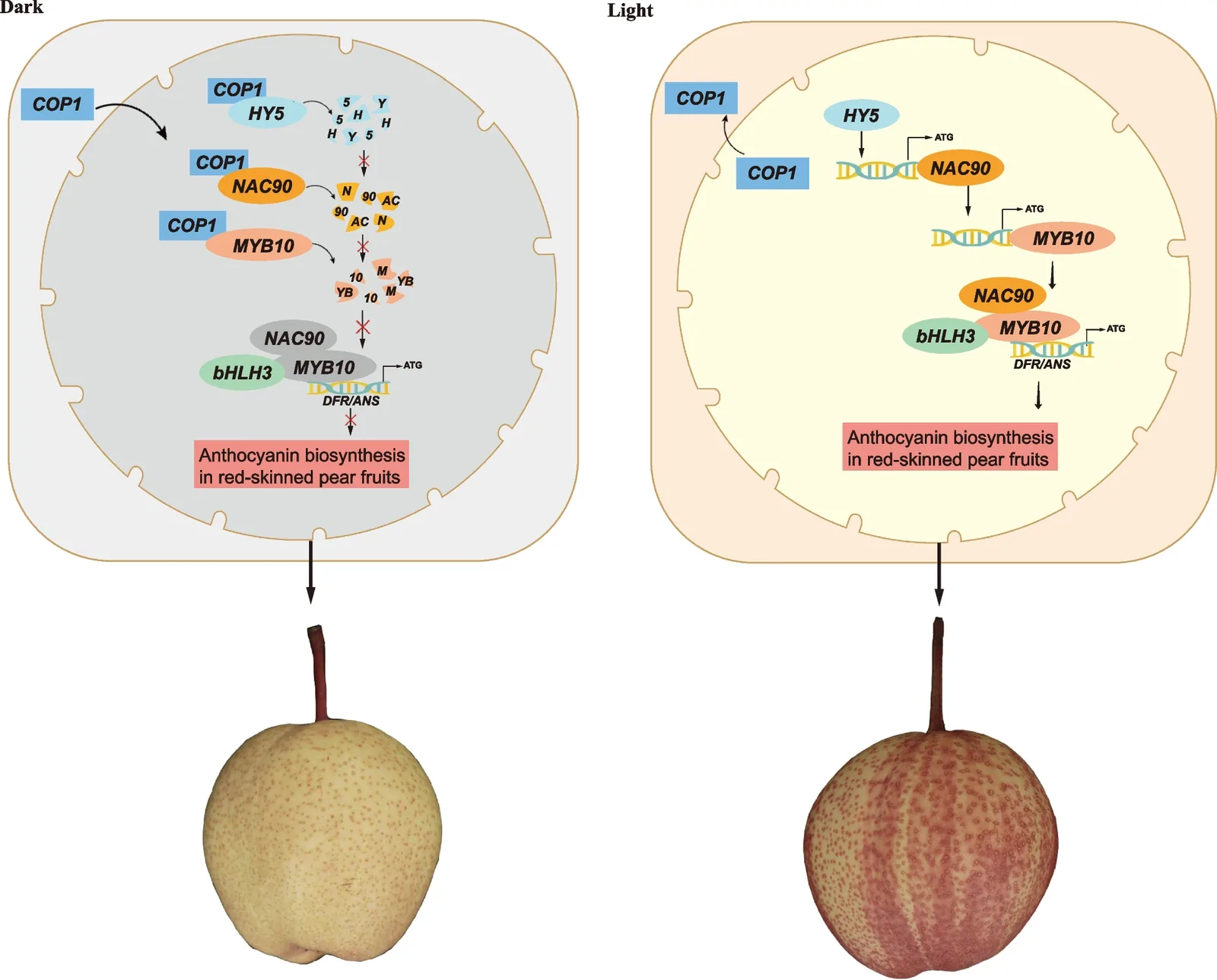
A proposed model for the regulation of anthocyanin biosynthesis by *PyNAC90* in red-skinned pear. Under dark conditions, PyCOP1 remains enzymatically active and translocates to the nucleus, where it mediates the degradation of PyHY5, PyMYB10, and PyNAC90. Consequently, PyHY5 fails to transcriptionally activate *PyNAC90* expression, and PyMYB10 also fails to interact with PyNAC90. This precludes the formation of the PyNAC90-PyMYB10-PybHLH3 regulatory complex required for anthocyanin biosynthesis, thereby suppressing pigment accumulation in pear fruits. Under light conditions, PyCOP1 protein activity is inhibited. After being activated by PyHY5, PyNAC90 in turn activates the *PyMYB10* promoter. Additionally, activated PyNAC90 interacts with the PyMYB10–PybHLH3 complex, synergistically promoting anthocyanin biosynthesis

Downstream of photoreceptors, the E3 ubiquitin ligase COP1/SPA acts as a central repressor of photomorphogenesis by targeting numerous positive regulators of light signaling, primarily transcription factors, for degradation in darkness. Under light conditions, COP1/SPA activity is suppressed, enabling light-responsive processes (Hoecker 2017). This regulatory mechanism is conserved across diverse plant species (Kang et al. 2022; Maier et al. 2013; Sun et al. 2024). In our study, PyCOP1 expression and activity gradually declined with increasing light exposure (Fig. 1C), consistent with its light-induced inhibition. We further found that PyCOP1 physically interacts with PyNAC90, reducing its transcriptional activation of *PyDFR* and *PyANS*, likely by promoting PyNAC90 degradation. Dual-luciferase assays confirmed this inhibitory effect, as co-expression of PyCOP1 with PyMYB10 and PyNAC90 significantly decreased promoter activity compared with PyMYB10 and PyNAC90 alone (Fig. 6D and E). A cell-free degradation assay confirmed that PyCOP1 mediates the degradation of PyNAC90 (Fig. 6F), thereby disrupting the PyNAC90-PyMYB10-PybHLH3 complex and attenuating its transcriptional activation of *PyANS* and *PyDFR*. Conversely, under light conditions, PyCOP1 activity is suppressed, allowing PyNAC90 to accumulate, stabilize the ternary complex, and promote anthocyanin biosynthesis in pear fruit skin (Fig. 7).

## Materials and methods

### Plant materials and growth conditions

Fruits of the pear cultivars ‘Zaosu’ and ‘Red Zaosu’ were collected from Liyuan Bay, Suqian City, Jiangsu Province, China. *Nicotiana tabacum* ‘*Samsun*’ and *N. benthamiana* plants were grown in a greenhouse under a 16–hour light/8–hour dark photoperiod at 22 ± 2 °C. Calli of the pear cultivar ‘Clapp’s Favorite’ were maintained on solid tissue culture medium at 22 ± 2 °C in complete darkness. At 35 days after full bloom (DAFB), fruits of ‘Mantianhong’ were enclosed in bags. These bags were removed 10 days prior to commercial maturity, re-exposing the fruits to sunlight. Random fruit sampling was performed at 4, 8, and 10 days following bag removal. Fruits that remained bagged served as controls (Liu et al. 2019).

### WGCNA analysis

A weighted gene co-expression network was constructed using the WGCNA R package (version 4.2.0) based on the gene expression matrix. An adjacency matrix was calculated from the FPKM values. The module eigengenes were correlated with the phenotypic data, specifically anthocyanin content. The modules most strongly associated with anthocyanin content were selected for subsequent analysis. The resulting co-expression network was visualized using Cytoscape (version 3.9.0). Genes from the antiquewhite2 and darkslateblue modules were then intersected with the set of differentially expressed genes (DEGs) that were upregulated in the de-bagged groups. DEGs were identified using the DESeq2 package. This intersection yielded a set of 80 candidate genes, from which PyNAC90 was identified.

### Genomic DNA extraction, RNA isolation, and RT–qPCR analysis

Genomic DNA (gDNA) was extracted using a Plant DNA Extraction Kit (Vazyme, China), and total RNA was isolated with a Plant Total RNA Kit (Vazyme, China). One microgram of RNA was reverse transcribed into first–strand cDNA using a HiScript II Reverse Transcriptase Kit (Vazyme, China), which includes a gDNA removal step. Quantitative real–time PCR (qRT–PCR) was performed in 20 μL reactions containing 2 × SYBR® Green Master Mix (ABclonal, USA) on a LightCycler96 system (Roche, Switzerland). Each run included a no–template control (NTC). Cycling conditions were: 95 °C for 30 s; 45 cycles of 95 °C for 3 s, 60 °C for 10 s, 72 °C for 30 s; final extension at 72 °C for 10 min. Relative transcript levels were calculated using the 2 ^-ΔΔCt^method (Kenneth And Thomas 2001), with three independent biological replicates, each with technical replicates. Primers were designed using NCBI Primer–BLAST for gene–specific amplification.

### Anthocyanin extraction and quantification

Anthocyanins were extracted from pear calli, fruit skins, and *N. tabacum* leaves following (Yao et al. 2017), with modifications. Approximately 100 mg of frozen tissue was homogenized in 0.1% HCl–methanol and incubated overnight at 4 °C in the dark. After centrifugation, supernatants were diluted fourfold in 0.1% HCl–methanol. Absorbance was measured at 530, 620, and 650 nm using a MAPADA UV–1800 spectrophotometer. Total anthocyanin content (TA, nmol·g⁻^1^) was calculated using the corrected absorbance [A = (A_530_ – A_620_) – 0.1 × (A_650_ – A_620_)] using the formula: TA = (A × V × 10⁶)/(ε × M), where V is the final volume (mL), M is sample fresh weight (g), and ε = 4.62 × 10^4^ L·mol⁻^1^·cm⁻^1^. Values represent the mean of three biological replicates.

### Transient expression in pear fruit and tobacco leaves

‘Red Zaosu’ pear fruits at ~105 DAFB and *N. tabacum* leaves were used for Agrobacterium–mediated transient transformation following (Yao et al. 2017) with minor modifications. Coding sequences of transcription factors were cloned into expression vectors: PyNAC90 into pSAK277 and TRV2; PyMYB10 and PybHLH3 into pSAK277, all under the 35S promoter. Constructs were transformed into *Agrobacterium tumefaciens* GV3101. Bacterial cultures were grown on selective media, resuspended in induction buffer (20 mM MgCl₂, 20 mM MES, pH 5.6, 150 μM acetosyringone) to OD₆₀₀ = 0.8–1.0, incubated for 2–3 h, and infiltrated into fruit and leaf tissues. Each treatment was performed in triplicate. Samples were kept in darkness for 24 h, followed by 4–7 days under light before phenotypic observation and sample collection.

### Generation of transgenic pear calli

Pear calli were generated from *Pyrus communis* ‘Clapp’s Favorite’ fruits via Agrobacterium–mediated transformation with GV3101 harboring 35S::PyNAC90–GFP. After selection on MS medium containing 20 mg L⁻^1^ hygromycin, positive calli were maintained in darkness at 22 ± 2 °C and subcultured biweekly. Integration was confirmed by RT–qPCR using *PyGAPDH* as the reference gene. Calli were treated with continuous white (15,000 lx) and blue light (1,600 lx) for 4–7 days, photographed, and harvested for further analysis.

### Subcellular localization

The *PyNAC90* coding sequence (without stop codon) was cloned into pCAMBIA1301–GFP to generate 35S::PyNAC90–GFP. Recombinant *Agrobacterium* cultures were prepared and infiltrated into 3– to 4–week–old *N. tabacum* leaves at OD₆₀₀ = 0.8, incubated for 2–3 h, and examined 3 dpi using a Leica confocal microscope.

### Dual‑luciferase assay

Promoters of *PyANS*, *PyDFR*, *PyMYB10*, and *PyNAC90* were amplified and cloned into pGreenII 0800–LUC, generating the reporter constructs proANS, proDFR, proMYB10, and proNAC90. Each reporter was transformed into *Agrobacterium tumefaciens* GV3101 carrying the pSoup helper plasmid. Reporter and effector plasmids were co–infiltrated into 3– to 4–week–old *N. benthamiana* leaves in the indicated combinations. At 3 dpi, leaf samples were collected, and Firefly (LUC) and Renilla (REN) luciferase activities were measured using the Dual–Luciferase® Reporter Assay System (Promega, USA) following the manufacturer’s protocol and established methods (Yao et al. 2017).

### Yeast two‑hybrid

Full–length *PyNAC90* and truncations (I–IV) were cloned into pGBKT7 (BD), and *PyMYB10* into pGADT7 (AD). Yeast strain AH109 was co–transformed with bait and prey constructs using the PEG/LiAc method with 10 μg salmon sperm DNA (Matchmaker™ GAL4 Two–Hybrid System, Clontech). Transformants were selected on SD/–Trp/–Leu at 28 °C for 2 days, then replica–plated onto SD/–Trp/–Leu/–Ade/–His and SD/–Trp/–Leu/–Ade/–His/X–α–Gal plates for 3–5 days to assess interaction–dependent growth and α–galactosidase activity.

### Luciferase complementary assay

*PyNAC90* and *PyMYB10* coding sequences were fused to Cluc and Nluc, respectively, to generate Cluc–PyNAC90 and PyMYB10–Nluc. At 3 dpi, *N. benthamiana* leaves were infiltrated with 1 mM luciferin potassium salt, incubated in darkness for 5 min, and imaged with a Tanon 5200 Multi-CCD system to detect luminescence.

### Bimolecular fluorescence complementation

PyNAC90 and PyMYB10 were cloned into 35S–pUC–SPYCE and 35S–pUC–SPYNE to generate PyNAC90–YCE and PyMYB10–YNE. Constructs were introduced into *A. tumefaciens* GV3101 and infiltrated into the abaxial epidermis of 3–week–old *N. benthamiana* leaves. At 3 dpi, 1 cm^2^ leaf sections were collected, mounted, and imaged using a Leica laser scanning confocal microscope to visualize fluorescence signals.

### Yeast one‑hybrid assay

The full–length *PyNAC90* and *PyHY5* CDS were fused to pB42AD (prey), while approximately 2 kb promoter regions of *PyNAC90*, *PyMYB10*, *PyANS*, and *PyDFR* were cloned into pLacZi (reporters). Assays were performed in *S. cerevisiae* EGY48 by co–transforming prey and reporter constructs, selecting transformants on SD/–Ura/–Trp, and evaluating β–galactosidase activity on X–Gal–containing plates, following a protocol analogous to Y2H.

### Cell-free protein degradation assay

The cell-free protein degradation assay was performed according to the method described by previous researchers (Zhao et al. 2024). Total proteins were extracted from tobacco leaves expressing GFP or PyCOP1-GFP using a plant protein extraction kit (Cwbio, China). Subsequently, the purified recombinant PyNAC90-GST protein was incubated at 25 °C with an equal amount of protein extracts containing GFP or PyCOP1-GFP in the presence of 1 mM ATP. Samples were collected at the indicated time points to assess PyNAC90-GST protein degradation in both groups. Additionally, MG132 (50 µM) was added to the two aforementioned combinations to further verify whether PyCOP1-mediated degradation occurs via the 26S proteasome pathway. PyNAC90-GST protein levels were detected using an anti-GST antibody. Protein quantification was performed using ImageJ (Schneider et al. 2012).

### Statistical analysis

All data were analyzed using Student’s *t*–test (*p < 0.05, **p < 0.01) with Microsoft Excel 2019 and GraphPad Prism 6.0.

## Supplementary Information

Supplementary Material 1: Fig. S1 Candidate genes identified by integrating differential expression analysis with key modules from WGCNA.** A** Gene clustering dendrogram and module assignment based on the topological overlap matrix in WGCNA. **B** The outer ring of the network represents transcription factors (TFs), while the inner ring represents pathway genes involved in anthocyanin biosynthesis. Nodes are labeled with gene names and colored based on correlation weight. TFs and pathway genes are connected via undirected edges. **C** Heatmap showing the correlations between the detected modules and anthocyanin content. **D** Volcano plots displaying differentially expressed genes at three stages between bagged and de-bagged fruits. **E** Overlap between differentially expressed genes and key WGCNA module genes. Fig. S2 Yeast two–hybrid and one‑hybrid assays of PyNAC90 interactions with anthocyanin–related transcription factors and structural gene promoters. **A–B** Yeast two-hybrid assay assessing interactions between PyMYB114 or PybHLH3 and the PyNAC90-II truncation variant. **C–E **Yeast one‑hybrid assay to detect the interaction between PyNAC90, and the *PyMYB10*, *PyANS* and *PyUFGT* promoters. The yeast cells grew on the SD/‑Trp/‑Ura and SD/‑Trp/‑Ura/ X‑Gal medium, respectively. **F **Yeast one‑hybrid assay to detect the interaction between PyHY5 and the *PyNAC90* promoter. The yeast cells grew on the SD/‑Trp/‑Ura/X‑Gal medium. Fig. S3 Expression pattern of PyNAC90 under dark conditions. **A** Expression levels of *PyNAC90* in ‘Red Zaosu’ pear skin under dark conditions. ‘Red Zaosu’ pear fruits were treated under light or dark conditions for 4, 8, and 10 days, after which the skin was collected for RT‑qPCR analysis. Samples exposed to light for 10 days served as controls. **B** Expression levels of *PyNAC90* in pear calli under dark conditions. The ‘Clapp’s Favorite’ calli were subjected to the same treatment as the ‘Red Zaosu’ pear fruits and then collected for RT‑qPCR analysis. Samples exposed to light for 10 days served as controls. Error bars denote mean ± SE of three biological replicates. ns: not significant; asterisks indicate statistically significant differences as determined by Student’s *t*‑test (* *P* < 0.05, ** *P* < 0.01). Fig. S4 Function of *PyNAC90* under dark conditions. **A** Transient overexpression of PyNAC90 in ‘Zaosu’ pear fruit. Photographed after 24 hours of dark treatment following transient injection. EV (empty vector pSAK277) was used as the control. OE, overexpression. The scale bar represents 1 cm. **B** Anthocyanin content in the skin of EV and PyNAC90-OE fruit. Samples were collected after 24 hours of dark treatment following transient overexpression. **C** Expression levels of anthocyanin-related genes in EV and PyNAC90-OE fruit skin. **D **Transient silencing of PyNAC90 in ‘Zaosu’ pear fruit. Photographed after 24 hours of dark treatment following transient injection. EV: empty vector (TRV1 + TRV2) control; VIGS: virus-induced gene silencing. The scale bar represents 1 cm. **E **Anthocyanin content in fruit skins of control and PyNAC90-VIGS fruit, measured by spectrophotometry. FW: fresh weight. **F **Expression levels of anthocyanin-related genes in control and PyNAC90-VIGS fruit skin, as analyzed by RT-qPCR. Error bars denote mean ± SE of three biological replicates. Asterisks indicate statistically significant differences as determined by Student’s *t*‑test (* *P* < 0.05, ** *P*< 0.01). Fig. S5 Specificity of the PyNAC90-VIGS construct. **A**. Neighbor-joining phylogenetic tree of PyNAC90 and its homologous genes in pear. The gene marked with a red star is PyNAC90. **B**. Expression levels of PyNAC90 homologous genes in pear skin samples with transient silencing of PyNAC90.

## Data Availability

The transcriptome data from a previously published bagging and debagging experiment used in this study are accessible on the Pear Genomics Database (PGDB): a comprehensive multi-omics research platform for *Pyrus* spp (https://pyrusgdb.sdau.edu.cn/download_data.html). The BioProject accession number is PRJ00002, and the BioSample accession numbers are SAM0000025 to SAM0000038. The data will be available from the corresponding author upon reasonable request.

## Abbreviations

FLCA: Firefly luciferase complementation assay
GFP: Green fluorescent protein FLUC Firefly luciferase
ORF: Open reading frame
RT‑qPCR: Reverse transcription quantitative real-time polymerase chain reaction
REN: Renilla luciferase
TFs: Transcription factors
VIGS: Virus–induced gene silencing
Y1H: Yeast one‑hybrid assay
Y2H: Yeast two‑hybrid assay
BiFC: Bimolecular fluorescence complementation
