# Supplementary material for: Light-responsive PyNAC90 orchestrates anthocyanin biosynthesis through combinatorial transcriptional activation and COP1-mediated degradation in pear
Source: Mol Hortic. 2026 Sep 10;6:74. doi: 10.1186/s43897-026-00254-7 (PMC13560111; doi:10.1186/s43897-026-00254-7)
Supplement: Supplementary file 1 [file 43897_2026_254_MOESM1_ESM.docx]

**Supplemental Figure:**

**Supplemental Figure S1 Candidate genes identified by integrating differential expression analysis with key modules from WGCNA**. **A** Gene clustering dendrogram and module assignment based on the topological overlap matrix in WGCNA. **B** The outer ring of the network represents transcription factors (TFs), while the inner ring represents pathway genes involved in anthocyanin biosynthesis. Nodes are labeled with gene names and colored based on correlation weight. TFs and pathway genes are connected via undirected edges. **C** Heatmap showing the correlations between the detected modules and anthocyanin content. **D** Volcano plots displaying differentially expressed genes at three stages between bagged and de-bagged fruits. **E** Overlap between differentially expressed genes and key WGCNA module genes.

**Supplemental Figure S**2 **Yeast two–hybrid and one‑hybrid assays of PyNAC90 interactions with anthocyanin–related transcription factors and structural gene promoters**. **A–B** Yeast two-hybrid assay assessing interactions between PyMYB114 or PybHLH3 and the PyNAC90-II truncation variant. **C–E** Yeast one‑hybrid assay to detect the interaction between PyNAC90 and the *PyMYB10*, *PyANS* and *PyUFGT* promoters. The yeast cells grew on the SD/‑Trp/‑Ura and SD/‑Trp/‑Ura/ + X‑Gal medium, respectively. **F** Yeast one‑hybrid assay to detect the interaction between PyHY5 and the *PyNAC90* promoters. The yeast cells grew on the SD/‑Trp/‑Ura/ + X‑Gal medium.

**Supplemental Figure 3** **Expression pattern of *PyNAC90* under dark conditions.** **A** Expression levels of *PyNAC90* in ‘Red Zaosu’ pear skin under dark conditions. ‘Red Zaosu’ pear fruits were treated under light or dark conditions for 4, 8, and 10 days, after which the skin was collected for RT‑qPCR analysis. Samples exposed to light for 10 days served as controls. **B** Expression levels of *PyNAC90* in pear calli under dark conditions. The ‘Clapp’s Favorite’ calli were subjected to the same treatment as the ‘Red Zaosu’ pear fruits and then collected for RT‑qPCR analysis. Samples exposed to light for 10 days served as controls. Error bars denote mean ± SE of three biological replicates. ns: not significant; asterisks indicate statistically significant differences as determined by Student’s *t*‑test (* *P* < 0.05, ** *P* < 0.01).

**Supplemental Figure 4** **Function of *PyNAC90* under dark conditions**. **A** Transient overexpression of *PyNAC90* in ‘Zaosu’ pear fruit. Photographed after 24 hours of dark treatment following transient injection. EV (empty vector pSAK277) was used as the control. OE, overexpression. The scale bar represents 1 cm. **B** Anthocyanin content in the skin of EV and PyNAC90-OE fruit. Samples were collected after 24 hours of dark treatment following transient overexpression. **C** Expression levels of anthocyanin-related genes in EV and PyNAC90-OE fruit skin. **D** Transient silencing of *PyNAC90* in ‘Zaosu’ pear fruit. Photographed after 24 hours of dark treatment following transient injection. EV: empty vector (TRV1 + TRV2) control; VIGS: virus-induced gene silencing. The scale bar represents 1 cm. **E** Anthocyanin content in fruit skins of control and PyNAC90-VIGS fruit, measured by spectrophotometry. FW: fresh weight. **F** Expression levels of anthocyanin-related genes in control and PyNAC90-VIGS fruit skin, as analyzed by RT-qPCR. Error bars denote mean ± SE of three biological replicates. Asterisks indicate statistically significant differences as determined by Student”s *t*‑test (* *P* < 0.05, ** *P* < 0.01).

**Supplemental Figure 5** **Specificity of the PyNAC90‑VIGS construct.** **A**. Neighbor‑joining phylogenetic tree of all NAC family members in the pear genome, with PyNAC90 marked by a blue star. **B** Neighbor‑joining phylogenetic tree of PyNAC90 and its homologous genes in pear where PyNAC90 is again indicated by a blue star. **C**. Expression levels of PyNAC90 homologous genes in pear skin samples with transient silencing of PyNAC90. Error bars denote mean ± SE of three biological replicates. ns: not significant; Asterisks indicate statistically significant differences as determined by Student’s *t*‑test (* *P* < 0.05, ** *P* < 0.01).

**Supplemental Table**

**Supplemental Table S1**

The promoter analysis of PyNAC90 indicated the light-responsive elements predicted by the PlantCARE database

| Motif | Strand | Distance from ATG | Sequance | Putative function |
| --- | --- | --- | --- | --- |
| AAAC-motif | - | 126 | CAATCAAAACCT | light responsive element |
| ACE | + | 90 | GCGACGTACC | cis-acting element involved in light responsiveness |
| AE-box | - | 433 | AGAAACAA | part of a module for light response |
|  | + | 840 | AGAAACTT | part of a module for light response |
| Box 4 | - | 1178 | ATTAAT | part of a conserved DNA module involved in light responsiveness |
|  | - | 1479 | ATTAAT | part of a conserved DNA module involved in light responsiveness |
|  | - | 1339 | ATTAAT | part of a conserved DNA module involved in light responsiveness |
| G-Box | + | 593 | CACGTT | cis-acting regulatory element involved in light responsiveness |
|  | + | 1724 | CACGTT | cis-acting regulatory element involved in light responsiveness |
| G-Box | - | 12 | CACGAC | cis-acting regulatory element involved in light responsiveness |
| TCT-motif | + | 144 | TCTTAC | part of a light responsive element |

**Supplemental Table S2**

Primers used in the study.

| Gene names | Forward primers | | | Reverse primers |
| --- | --- | --- | --- | --- |
| PyNAC90(qRT) | ACAAGCTCGAAGGGAAGCAA | | | TCGTTGTTGCCTCGGTGTAA |
| PyActin(qRT) | TGAGACATTCAACACCCCGGCTAT | | | GATGGCATGTGGGAGGGCATA |
| PyCHSA(qRT) | TCTTGTTGTGTGCTCTGAGATCACC | | | GATAGTTTGGGCCGCAGACACCAGC |
| PyCHI(qRT) | AGATCAAAACTTCCCACCGGGC | | | CAGCTTCAGGCACGGATGCA |
| PyF3H(qRT) | GCTGTCCATAGCCACATTCCAGAA | | | CATCTCGGTGTAGGTGATTGGCG |
| PyDFR(qRT) | TGGTATCATCAAGCAGGGCCAGTAT | | | CGTGTGACGAACAAATGTAGCGG |
| PyANS(qRT) | AGGGGCATGGTGAACAAGGAAA | | | AGTTCGTGGTGGGAACATTGCC |
| PyUFGT(qRT) | CCTTCCCTTTTGCCACTCA | | | CAGCCACATCGTACACCCTTA |
| PyMYB10(qRT) | GACCAATGTGATAAGACCTCAGCC | | | CCGTTCTTTGTTGACGACGAC |
| PyMYB114(qRT) | GCCACATCCGTCATAAGACCTC | | | GCCACTCATGTGTAACCCTTC |
| PybHLH3(qRT) | TTGTGGAGGGAAGTGGCGGT | | | AGCTCCCTAAGTGTTTGCATCAC |
| PyNAC90-277- | actagtggatccaaagaattcATGGCTTCCTTACCAAACACAGA | | | agaagtactctcgagaagcttGAGGAAATTTAGTTGTTCCCATTCC |
| PyMYB10-277- | actagtggatccaaagaattcATGGAGGGATATAACGTTAACTTGAG | | | agaagtactctcgagaagcttCTATTCTTCTTTTGAATGATTCCAAAG |
| PybHLH3-277- | actagtggatccaaagaattcATGGCTGCACCGCCGCCA | | | agaagtactctcgagaagcttTTAAGAGTCAGATTGGGGTATAATTTG |
| PyCOP1-277)- | actagtggatccaaagaattcATGGAGGAGTGCTCGACCG | | | agaagtactctcgagaagcttCTATGCCGCAAGAACCAACAC |
| proDFR-0800 | gtcgacggtatcgataagcttAAATTTCCTAAAAAATATACTCGTGT | | | cgctctagaactagtggatccCTTATAAAATATTTATAGTGGGGGGC |
| proANS-0800 | gtcgacggtatcgataagcttAGGGCTTTCTTTTATCATGATCTAAGA | | | cgctctagaactagtggatccTTTTGGAGCTGGCTTTCGAC |
| proMYB10-0800 | ttcctgcagcccgggggatccAGTTAGTAGAATTATCTAAATATCTCTAACATGATG | | | cgctctagaactagtggatccCTCGTATCTGCTAGCAGCTAAGCT |
| proNAC90-0800 | ttcctgcagcccgggggatccATGCTATATCGAAGAGGAAGGAAGC | | | cgctctagaactagtggatccTATATTTGAGTTTGGTATCTCTGTGTTTG |
| PyCOP1-GFP | gagaacacgggggactctagaATGGGAGAGAGCTCAATGGGT | | | gcccttgctcaccatggatccGGCTGCAAGTACGAGCACTTTA |
| PyNAC90-GFP | gagaacacgggggactctagaATGGCTTCCTTACCAAACACAGA | | | gcccttgctcaccatggatccGAGGAAATTTAGTTGTTCCCATTCC |
| PyNAC90-BK | atggccatggaggccgaattcATGGCTTCCTTACCAAACACAGA | | | ccgctgcaggtcgacggatccTTAGAGGAAATTTAGTTGTTCCCATTC |
| PyNAC90-Ⅰ-BK | | | atggccatggaggccgaattcATGGCTTCCTTACCAAACACAGA | ccgctgcaggtcgacggatccTTATGGTAGCTCCTCCATGTTTTTT |
| PyNAC90-Ⅱ-BK | | | atggccatggaggccgaattcATGGCTTCCTTACCAAACACAGA | ccgctgcaggtcgacggatccTTAGGCTCTGTACACATTCATCTTCC |
| PyNAC90-Ⅲ-BK | | | atggccatggaggccgaattcATGATTGAAGTAGACAACTTAACTAGCACT | ccgctgcaggtcgacggatccTTAGAGGAAATTTAGTTGTTCCCATTC |
| PyNAC90-Ⅳ-BK | | | atggccatggaggccgaattcATGGCTTCCTTACCAAACACAGA | ccgctgcaggtcgacggatccTTACGGTGTAAAGAAAAACCATTGC |
| PyMYB10-AD | atggccatggaggccgaattcATGGAGGGATATAACGTTAACTTGAG | | | ccgctgcaggtcgacggatccCTATTCTTCTTTTGAATGATTCCAAAG |
| PyMYB1140-AD | atggccatggaggccgaattcATGAGGAAGGGTGCCTGG | | | ccgctgcaggtcgacggatccCTAAATCTTAGTTATCTCTTCTTC |
| PybHLH30-AD | gccatggaggccagtgaattcATGGCTGCACCGCCGCCA | | | cagctcgagctcgatggatccTTAAGAGTCAGATTGGGGTATAATTTG |
| PyCOP10-AD | gccatggaggccagtgaattcATGGAGGAGTGCTCGACCG | | | cagctcgagctcgatggatccCTATGCCGCAAGAACCAACAC |
| PyNAC90-Cluc | | tacgcgtcccggggcggtaccATGGCTTCCTTACCAAACACAGA | | tgtagtccatttgttggatccGAGGAAATTTAGTTGTTCCCATTCC |
| PyCOP1-Nluc | | gagctcggtacccggggatccATGGAGGAGTGCTCGACCG | | cgcgtacgagatctggtcgacCTATGCCGCAAGAACCAACAC |
| PyMYB10-Nluc | | ctcggtacccggggatccATGAAGATTCAGTGTGATGTGT | | gtacgagatctggtcgacAGAGTCAGATTGGGGTATAATTTG |
| PyNAC90-YCE | | gagaacacgggggactctagaATGGCTTCCTTACCAAACACAGA | | gacagtactatcgatggatccGAGGAAATTTAGTTGTTCCCATTCC |
| PyCOP1-YNE | | gagaacacgggggactctagaATGGAGGAGTGCTCGACCG | | gacagtactatcgatggatccCTATGCCGCAAGAACCAACAC |
| PyMYB10-YNE | | gagaacacgggggactctagaATGGAGGGATATAACGTTAACTTGAG | | gacagtactatcgatggatccCTATTCTTCTTTTGAATGATTCCAAAG |
